# Pharmacokinetics of Tolfenamic Acid Administered Orally and Intravenously at Different Doses in Pekin Ducks (*Anas platyrhynchos domestica*)

**DOI:** 10.3390/ani15162326

**Published:** 2025-08-08

**Authors:** Orhan Corum, Kamil Uney, Pedro Marin, Duygu Durna Corum, Devran Coskun, Elena Badillo, Muammer Elmas

**Affiliations:** 1 Department of Pharmacology and Toxicology, Faculty of Veterinary Medicine, University of Hatay Mustafa Kemal, Hatay 31060, Türkiye; ddurnacorum@gmail.com; 2 Department of Pharmacology and Toxicology, Faculty of Veterinary Medicine, University of Selcuk, Konya 42031, Türkiye; kuney@selcuk.edu.tr (K.U.); melmas@selcuk.edu.tr (M.E.); 3 Department of Pharmacology, Faculty of Veterinary Medicine, University of Murcia, 30100 Murcia, Spain; ebp2@um.es; 4 Department of Pharmacology and Toxicology, Faculty of Veterinary Medicine, University of Siirt, Siirt 56100, Türkiye; devrancoskun@gmail.com

**Keywords:** bioavailability, ducks, NSAIDs, pharmacokinetics, plasma protein binding, tolfenamic acid

## Abstract

This study aimed to examine the pharmacokinetic changes in tolfenamic acid administered intravenously and orally to ducks at different doses (2, 4, and 8 mg/kg). Furthermore, the binding ratio to plasma proteins was assessed utilizing the ultrafiltration method. Eighteen male Pekin ducks were randomly assigned to three dosage groups (2, 4, and 8 mg/kg), with each group undergoing a trial in two phases: intravenous (IV) and oral administration. The sample was analyzed using an approved HPLC-UV method. A non-compartmental analysis was utilized to evaluate the pharmacokinetic data. For 2 mg/kg IV injection, the area under the curve from zero to infinity (AUC_0-∞_), total clearance (Cl_T_), volume of distribution at steady state (V_dss_), and elimination half-life (t_1/2ʎz_) were 13.03 h*µg/mL, 0.15 L/h/kg, 0.30 L/kg, and 1.72 h, respectively. Following oral administration at a dose of 2 mg/kg, the AUC_0-∞_, peak plasma concentration (C_max_), and bioavailability were 6.32 h*µg/mL, 2.25 µg/mL, and 48.52%, respectively. The t_1/2ʎz_ was extended, C_max_ and AUC_0-∞_ elevated, T_max_ shortened, and Cl_T_ decreased in a dose-dependent manner. No dose-related change was observed in V_dss_ and bioavailability. In ducks, tolfenamic acid’s plasma protein binding was 99.74%, unaffected by concentration. These results may contribute to the application of tolfenamic acid in ducks at different doses, but dose-related changes in therapeutic efficacy should also be demonstrated.

## Simple Summary

This study examined how tolfenamic acid behaves in Pekin ducks when given either intravenously or orally at different doses. The key findings showed that with increasing doses, the drug’s clearance reduced, and its elimination half-life extended, while the volume of distribution and bioavailability stayed the same across doses. The drug also demonstrated a high level of plasma protein binding, consistent across concentrations. These insights are critical for maximizing the drug’s effectiveness and reducing side effects, as higher doses might affect the elimination process. The results suggest that using higher doses could extend the drug’s anti-inflammatory and analgesic effects, highlighting the importance of fine-tuning doses to improve treatment outcomes. Future research should focus on how the drug distributes to inflammation sites and its safety profile to further optimize dosing strategies.

## 1. Introduction

Tolfenamic acid, also known as N-(2-methyl-3-chlorophenyl)-anthranilic acid, is a non-steroidal anti-inflammatory drug (NSAID) belonging to the fenamate class. This medicine was initially utilized in humans and subsequently in animals because of its antipyretic, anti-inflammatory, and analgesic properties [1,2]. It also possesses antiendotoxic, antibacterial, and anticancer properties [3,4]. It exerts its effect by inhibiting cyclooxygenase (COX)-1 and COX-2, the enzymes responsible for the generation of prostaglandins (PGs) and thromboxanes (TXs). Its selectivity for COX enzymes in birds remains unclear; however, it has non-selective activity in calves and COX-2 selective activity in goats [5,6]. Tolfenamic acid is approved for use in dogs, cats, pigs, and cattle by the European Medicines Agency [2].

The absence of knowledge regarding pain in birds makes it hard to choose an effective painkiller. Pain behaviors vary depending on duration (acute and chronic pain), species, and individual birds, making it difficult to define [7]. However, if an operation or injury results in tissue damage accompanied by alterations in posture, temperament, or behavior, the bird can be assumed to be in pain [8]. Birds, like mammals, possess the neuroanatomical and neuropharmacological pathways required for pain perception, and COX enzymes contribute to the pathophysiology of pain and inflammation [7,9]. NSAIDs are indicated for treating severe injuries, arthritis, lameness, and preoperative and postoperative discomfort in birds [10,11].

Ducks are a significant bird species in the Anatidae family that have valuable products such as eggs, meat, and feathers [12]. It was reported that ducks constituted approximately 3% of the world’s poultry population in 2020 [13]. Ducks are raised in many regions of the world, particularly in China, Bangladesh, Vietnam, and Indonesia, due to their advantages, such as ease of care, easy adaptation to environmental conditions, and resistance to diseases [13,14]. The Pekin duck is an important duck breed that has gained worldwide fame for its soft meat and high-quality eggs [15] and is widely raised in our country. Pekin ducks have a long lifespan and are popular as pets because they are extremely durable, affordable, and simple to care for. Painkillers can be used in commercial and pet ducks for pre- and postoperative pain, degenerative joint diseases and musculoskeletal pain, and bacterial infection cases [8].

Tolfenamic acid is not licensed for use in bird species; nonetheless, data exists regarding its pharmacokinetics following administration at a dose of 2 mg/kg in ducks [8], as well as the effect it has on biochemical parameters at doses ranging from 2 to 8 mg/kg [14]. The efficacy of tolfenamic acid is dose-dependent, and its effects increase with increasing dose in calves and dogs [4,16]; similarly, its pharmacokinetics also change with increasing dose in sheep and goats [17,18]. The area under the curve and elimination half-life of this NSAID increased, and total clearance decreased, contingent upon the dosage in sheep and goats [1,17,18]. This change in the pharmacokinetic parameters may lead to a longer dosing interval and thus reduce the frequency of administration when used repeatedly, which is highly beneficial in veterinary medicine. This study aimed to examine the pharmacokinetic alterations of tolfenamic acid administered orally and intravenously at different doses (2, 4, and 8 mg/kg) in Pekin ducks and to evaluate the binding ratio to plasma proteins in this avian species.

## 2. Materials and Methods

### 2.1. Animals

This study utilized eighteen healthy male Pekin ducks, with weights ranging from 2.10 to 2.85 kg (mean weight: 2.46 ± 0.22 kg). The ducks were acquired from a local farm in Hatay, Türkiye, and were equipped with numbered leg rings for efficient identification. Ducks were assessed to be in excellent health based on anamnesis, behavioral monitoring, and physical examination. The ducks were housed in pens with an open space for ten days prior to the experimental investigation to acclimate to the environment. Ducks were fed with a drug-free pelleted diet twice daily, and water was provided ad libitum. Hatay Mustafa Kemal University’s Local Ethics Committee for Animal Research Studies authorized (2022/03-16) the study protocol.

### 2.2. Experimental Design

The ducks were arbitrarily allocated into three groups, each receiving a different dosage. The experimental investigation for each group was conducted in two phases, intravenous and oral, using a longitudinal design after a 15-day drug washout interval. In the initial stage, tolfenamic acid (Tolfenak 40 mg/mL, Alke, Istanbul, Türkiye) was administered intravenously at a dose of 2 mg/kg to group 1, 4 mg/kg to group 2, and 8 mg/kg to group 3. In the second phase, the groups received the same doses orally. Intravenous (IV) injection was conducted into the right brachial vein, and oral administration was performed using crop gavage. Blood samples (~0.4 mL) were obtained at 17 distinct time points (0, 0.08, 0.25, 0.5, 0.75, 1, 1.5, 2, 3, 4, 6, 8, 10, 12, 24, 36, and 48 h) following IV and oral administration. A catheter (24-gauge catheter) inserted into the left brachial vein was utilized for blood collection during the initial 12 h. In the following blood collections, venipuncture was performed on both brachial veins. To ensure patency, the catheter was washed with 0.5 mL of sterile saline solution (0.9%) and heparin (10 IU/mL) at each collection time point. In each blood collection, the initial 100 µL of blood was discarded. Plasma samples were obtained by centrifuging the blood at 4000× *g* within one hour and stored at −80 °C until analysis. Tolfenamic acid analysis in plasma samples was performed within three months.

### 2.3. HPLC Conditions

The equipment information of the HPLC system was reported in the previous study [1]. Shimadzu Corp.’s LC Solution software (Version 1.25 SP5) was used for data analysis. The injection loop volume was established at 20 μL. The chromatographic separation was conducted using an Inertsil ODS-3 column (4.6 × 250 mm; 5 μm) at 40 °C. The mobile phase consisted of 65% acetonitrile and 35% orthophosphoric acid in water (0.1%) with a flow rate of 1 mL/min. The quantification wavelength was determined to be 289 nm.

### 2.4. Validation of the Analytical Method

The quantitative HPLC method was completely verified for each of the duck plasmas in terms of precision, recovery, accuracy, and linearity, according to the EMA guidelines [19]. Tolfenamic acid (98.0%, Sigma-Aldrich, St. Louis, MO, USA) stock solutions (1 mg/mL) and all associated dilutions were prepared in a solvent combination containing 40% methanol, 40% acetonitrile, and 20% water. The stock solution or working standard was added to blank duck plasma to produce calibration standards (0.04–80 μg/mL) and quality control samples (0.1, 4, and 40 μg/mL). The analytical method of tolfenamic acid exhibited linearity with an R^2^ of >0.998. The limit of detection (LOD) was 0.02 μg/mL, and the lower limit of quantification (LOQ) was 0.04 μg/mL. The analysis of quality control samples was conducted six times over three separate days to determine recovery, precision, and accuracy. The mean recovery in duck plasma exceeded 94%. The intraday precision CV% value was 8.32%, and the interday precision CV% value was 7.01%. The intraday bias and the interday bias were ± 3.15% and ± 2.44%, respectively.

### 2.5. Sample Extraction

The analysis of tolfenamic acid in plasma samples was conducted following the previously published method [1,8]. Acetonitrile (150 μL) was added to the plasma samples (100 μL) to denature the proteins. Then, vortexing (45 s) and centrifuging (10,000× *g* for 15 min) were performed. The upper layer was placed in autosampler vials, and 20 μL was injected into the system.

### 2.6. Pharmacokinetic Analysis

The data acquired from HPLC analysis for each duck were recorded in an Excel file, and plasma concentrations were calculated. The WinNonlin program version 6.1.0.173 was employed to ascertain pharmacokinetic data by non-compartmental analysis. The abbreviations and definitions of pharmacokinetic parameters are presented in the footnote of Table 1. The λ_z_ was determined by the absolute values from more than three data points using a log-linear regression analysis. The t_1/2λz_ was determined using the formula t_1/2λz_ = 0.693/λ_z_. The AUC was calculated using the linear log trapezoidal method for IV administration and the linear-up log-down method for oral administration. The plasma concentration–time curve was directly observed to determine the C_max_ and T_max_ for oral delivery. The bioavailability (F) was determined as the percentage ratio of the AUC following oral administration to that following IV administration. The body extraction ratio (E_body_) for IV injection was established as previously documented [20].

### 2.7. Plasma Protein Binding

The ultrafiltration technique was employed to ascertain the plasma protein binding of tolfenamic acid, as previously documented [21]. Tolfenamic acid was added to drug-free plasma samples from ducks at four different concentrations (0.4, 2, 10, and 40 μg/mL). These concentrations were studied in triplicate. The mixture was incubated at 41 °C for 30 min to allow the binding to reach equilibrium. Subsequently, 1 mL of the sample was transferred to Amicon Ultra Centrifugal Filters (Merck Milipore Ltd., Tullagreen, Carrigtwohill, Ireland) and then centrifuged (4000× *g* for 15 min). The ultrafiltrate obtained after centrifugation was analyzed directly by HPLC to determine free tolfenamic acid concentrations. Additionally, extraction was performed for concentrations of 0.4, 2, 10, and 40 μg/mL to determine the total drug concentration and then analyzed by HPLC. The proportion of plasma protein binding was assessed using the following formula: Protein binding (%) = [100 * (total drug-free drug)/total drug]. A preliminary investigation was undertaken to analyze the nonspecific interaction between tolfenamic acid and the ultrafiltration system. The results indicated that binding was negligible, with under 1% of the medication connecting with the device.

### 2.8. Statistical Analysis

The statistical analysis was conducted using SPSS version 22.0. A *p*-value below 0.05 was deemed statistically significant. Pharmacokinetic data are displayed as geometric mean (min-max) except for T_max_, which was presented as median (min-max). Levene’s test assessed variance homogeneity, whereas the Shapiro–Wilk test evaluated data distribution normality. The pharmacokinetic parameters and plasma protein binding ratio were assessed using one-way analysis of variance (ANOVA) and the post hoc Tukey test. Differences between oral and IV groups at the same dose level were examined with a paired *t*-test.

## 3. Results

### 3.1. Animals

No signs of toxicity (neurological and behavioral abnormalities) due to tolfenamic acid were seen in ducks after IV and oral administration of three dose levels. The ducks’ attitude, appetite, and activity levels were normal throughout the study and afterwards (up to 1 week).

### 3.2. Intravenous Administration

**Figure 1:**
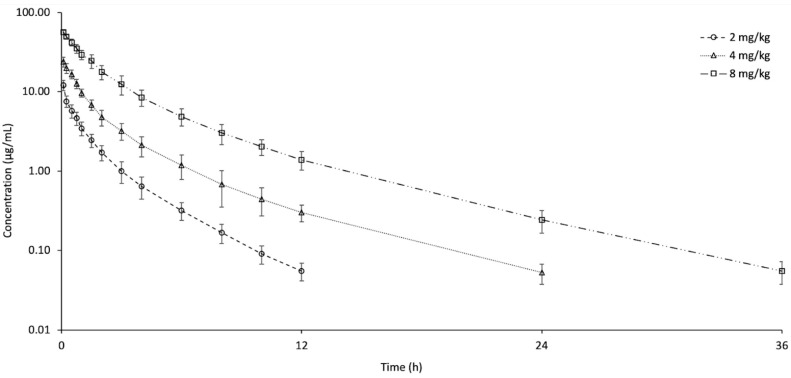
Semi-logarithmic plasma concentration–time curves of tolfenamic acid following single intravenous administration at doses of 2, 4, and 8 mg/kg in Pekin ducks (*n* = 6, mean ± SD).

Figure 1 shows a semi-logarithmic plot of the plasma concentrations (mean ± SD) of tolfenamic acid after intravenous injection of 2, 4, and 8 mg/kg doses. Tolfenamic acid was identified in plasma for periods of 12, 24, and 36 h following intravenous injections of 2, 4, and 8 mg/kg, respectively. The summary of pharmacokinetic parameters after IV administration is listed in Table 1. For the 2 mg/kg dose, AUC_0-last_, t_1/2ʎz_, V_dss_, and Cl_T_ were 12.90 h*µg/mL, 1.72 h, 0.30 L/kg, and 0.15 L/h/kg, respectively. Significant changes were seen in these parameters except V_dss_ at 4 and 8 mg/kg doses compared to 2 mg/kg. Simultaneously with the dose escalation, t_1/2ʎz_ was extended, dose-normalized AUC_0-last_ increased, and Cl_T_ reduced. No dose-related change was observed in V_dss_. The E_body_ value of tolfenamic acid diminished in accordance with the dose.

### 3.3. Oral Administration

**Figure 2:**
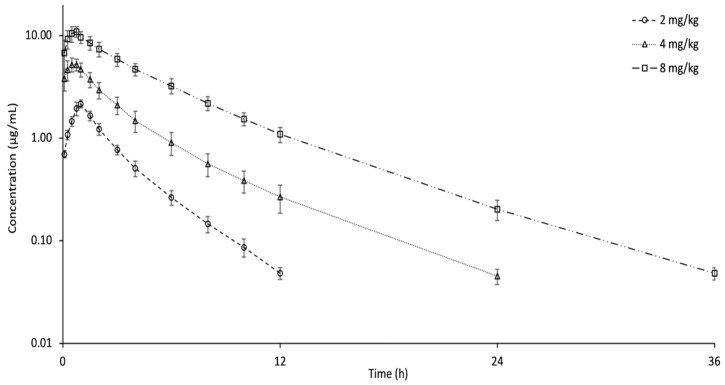
Semi-logarithmic plasma concentration–time curves of tolfenamic acid following single oral administration at doses of 2, 4, and 8 mg/kg in Pekin ducks (*n* = 6, mean ± SD).

Figure 2 shows a semi-logarithmic plot of the plasma concentrations (mean ± SD) of tolfenamic acid after oral administration of 2, 4, and 8 mg/kg doses. Tolfenamic acid was identified in plasma for periods of 12, 24, and 36 h following oral doses of 2, 4, and 8 mg/kg, respectively. The summary of pharmacokinetic parameters after oral administration is listed in Table 1. For the 2 mg/kg dose, t_1/2ʎz_, AUC_0-last_, C_max_, T_max_, and bioavailability were 2.13 h, 6.18 h*µg/mL, 2.25 µg/mL, 1.00 h, and 48.52%, respectively. The t_1/2ʎz_ and AUC_0-last_ increased with increasing dose. The 4 and 8 mg/kg dosages led to an elevation in C_max_ and a shortening of T_max_ relative to the 2 mg/kg dose. No dose-related change was observed in bioavailability. In comparison to IV treatment, the t_1/2ʎz_ was longer and the AUC was lower in all dose groups after administration via the oral route.

### 3.4. Plasma Protein Binding

The binding ratio of tolfenamic acid to plasma protein is displayed in Table 2. The in vitro protein binding ratio in duck plasma ranged from 99.56 ± 0.08% to 99.85 ± 0.02% with an average of 99.74 ± 0.20%. It was also established that binding to plasma proteins was independent of concentration.

## 4. Discussion

Despite the prevalence of pain- and inflammation-related disorders in ducks, their use remains limited due to the absence of approved NSAIDs. Therefore, pharmacokinetic and therapeutic studies of NSAIDs in these species contribute to their extra-label use. Although the pharmacokinetics of tolfenamic acid have been established in ducks [8], partridges [20], and geese [22], no research on dose-dependent pharmacokinetic variations in bird species has been found. This study is the first document showing the dose-dependent pharmacokinetics of tolfenamic acid in ducks. These results may contribute to the use of different doses of tolfenamic acid in the treatment of pain and inflammation in these animals.

The therapeutic effect of tolfenamic acid increases dose-dependently. In a comparison of the efficacy of 2, 4, and 8 mg/kg doses of tolfenamic acid on the suppression of PGE_2_ and TXB_2_ in dogs and calves, it was shown that efficacy increased with dose, peaking at the 8 mg/kg dose [4,16]. The IV administration of tolfenamic acid at a dose of 2–8 mg/kg in ducks did not induce significant alterations in biochemical parameters [14]. Tolfenamic acid was well tolerated in goats at doses of 2 and 4 mg/kg [18] and in sheep at doses of 2–8 mg/kg [17]; however, it induced neurological problems in sheep at 16 mg/kg and in cattle at 18–20 mg/kg [2,17]. The dosages employed in this investigation were determined by taking into account these reported efficacy and safety studies.

The average plasma protein binding for tolfenamic acid in ducks was 99.74%, and this binding was concentration-independent. The value in ducks was comparable to that reported in rainbow trout (99.48%), dogs (99%), and calves (92%) [21,23,24]. However, it was significantly higher than the 19–31% in turtles and 26% in crocodiles [25,26,27]. It has been suggested that the low levels of plasma albumin in these species may result in low plasma protein binding [25].

Following the IV administration in ducks at the 2 mg/kg dose, the V_dss_ was 0.30 L/kg, which was consistent with the value previously reported (0.25–0.41 L/kg) in ducks, geese, and chukar partridges [8,20,22]. Generally, NSAIDs exhibit a high affinity for plasma proteins, leading to low volumes of distribution [5]. Since tolfenamic acid is highly (99.74%) bound to plasma proteins in ducks, the V_dss_ value may be low. No difference in V_dss_ was observed after IV administration of 4 and 8 mg/kg compared to 2 mg/kg; similarly, no dose-dependent changes were seen in goats [18]. However, in sheep, the V_dss_ value changed based on the dose administered [1,17]. Dose-dependent alterations in plasma protein binding may result in a change in V_dss_. However, in ducks, protein binding of tolfenamic acid was independent of concentration.

The current investigation determined that the Cl_T_ of tolfenamic acid at a dose of 2 mg/kg was 0.15 L/h/kg, consistent with findings (0.14–0.16 L/h/kg) in ducks, geese, and partridges [8,20,22]. The E_body_ value for the same dose of tolfenamic acid in ducks was 0.011, indicating a low extraction ratio (low E_body_ = 0.05) [28], as previously reported in ducks (0.010) [8], geese (0.014) [22], and partridges (0.016) [20]. In comparison to 2 mg/kg, the Cl_T_ and E_body_ values of tolfenamic acid diminished dose-dependently at other doses. Likewise, prior research has indicated that the clearance of tolfenamic acid diminishes in a dose-dependent manner [1,17,18]. Tolfenamic acid undergoes significant hepatic metabolism and is eliminated in urine and bile as metabolites. Because metabolism differs between animal species, the percentage excreted unaltered in urine varies (1–89%) [2,29]. No data exists about the metabolism and excretion of tolfenamic acid in avian species. Therefore, the dose-dependent decrease in Cl_T_ may be due to saturation of hepatic metabolism or excretion pathways. Furthermore, NSAIDs work by inhibiting PG production, which is crucial for renal hemostasis and renal perfusion [30]. The decrease in renal blood flow due to PG inhibition with increasing doses of tolfenamic acid may contribute to the decrease in Cl_T_.

The volume of distribution, biotransformation, and excretion of drugs may differ between birds and mammals due to differences in body weight and surface area, body temperature, body composition, glomerular filtration rate, and metabolic pathways [31]. The pharmacokinetic parameters of tolfenamic acid at 2 mg/kg dose in ducks, such as t_1/2ʎz_ (1.72 h), Cl_T_ (0.15 L/h/kg), and V_dss_ (0.30 L/kg), exhibited considerable variability but overall suggest patterns similar to those (t_1/2ʎz_; 1.57–6.09 h, Cl_T_; 0.07–0.30 L/h/kg, and V_dss_; 0.30–0.68 L/kg) observed at 2 mg/kg dose in mammals [22,32]. Specifically, the data indicate that in ducks, tolfenamic acid displays rapid elimination, a relatively small volume of distribution, and a moderate clearance rate, aligning more closely with the pharmacokinetic profiles of certain mammalian species such as sheep and goats, characterized by quick drug elimination, limited tissue distribution, and intermediate clearance rates [17,18].

The observed C_max_ in the ducks at an oral dose of 2 mg/kg (2.25 µg/mL at 1.00 h) was consistent with the values reported in ducks (2.23 µg/mL) [8] and geese (2.92 µg/mL) [22]. In comparison to the 2 mg/kg, the 4 and 8 mg/kg oral doses exhibited a greater C_max_ (dose-normalized) and a shorter T_max_. There is no study evaluating C_max_ after oral administration of tolfenamic acid at different doses. However, no difference was noted in C_max_ value in dogs and calves after IM administration of different doses [4,16], while an increase was reported in crocodiles [25]. The Cl_T_, V_d_, and bioavailability influence the determination of the C_max_ value [22]. In ducks, V_d_ and bioavailability did not alter with dosage, but Cl_T_ was reduced; hence, C_max_ may have increased due to the decrease in Cl_T_ at high doses. The bioavailability in the ducks at an oral dose of 2 mg/kg (48.52%) was consistent with the values reported in ducks (43.43%) [8] but lower than that recorded in geese (76.03%) [22]. No difference in bioavailability was observed between dose groups. AUC was observed to increase dose-dependently after both IV and oral administration.

The t_1/2ʎz_ value after oral administration at different doses was longer than that after IV injection. Similarly, longer t_1/2ʎz_ after oral administration compared to IV has been reported in ducks, geese, and sheep [8,17,22]. Some studies have shown that the extended t_1/2ʎz_ following extravascular application may be attributed to the flip-flop phenomenon [4,17]. The MAT value in the flip-flop phenomenon has to be longer than the MRT_IV_ [33]. When the MAT value was studied, it was shown that the long t_1/2ʎz_ for oral administration in ducks was not caused by the flip-flop phenomenon. This might be attributed to another condition (gastric emptying, small intestinal transit time, stomach contents, physicochemical properties of the drug) impacting the absorption process; however, further research is required to ascertain this.

Pharmacodynamic investigation on the therapeutic properties of tolfenamic acid in bird species is insufficient. The IC_50_ values of tolfenamic acid for exudate PGE_2_ and plasma TxB_2_ in mammals were 0.07–0.23 μg/mL and 0.14–1.3 μg/mL, respectively [5,6,16]. These data were used to estimate the pharmacodynamic efficacy of tolfenamic acid in ducks. The IC_50_ values required for PGE_2_ inhibition were obtained up to 6, 12, and 24 h after administration of 2, 4, and 8 mg/kg doses by the IV route, respectively, while they were obtained up to 6, 12, and 12 h after administration of 2, 4, and 8 mg/kg doses by the oral route, respectively. The IC_50_ values required for TxB_2_ inhibition were obtained up to 2, 4, and 12 h after administration of 2, 4, and 8 mg/kg doses by the IV route, respectively, while they were obtained up to 1.5, 4, and 12 h after administration of 2, 4, and 8 mg/kg doses by the oral route, respectively. This evaluation, which was made using the plasma concentrations obtained in this study and the reported IC_50_ values, showed that the duration of effect may increase as the dose increases in both the IV and oral routes. However, previous studies have reported that plasma concentrations may not reflect the efficacy of tolfenamic acid because concentrations and pharmacokinetic data in plasma and exudate are different [4,5,6,16]. Also, it should be kept in mind that IC_50_ values obtained from mammals were used to evaluate therapeutic efficacy and cannot be directly generalized to birds. Therefore, further studies are needed to examine the pharmacokinetic/pharmacodynamic relationship after administration of different doses of tolfenamic acid to ducks.

Drugs may produce residues in food products generated from animals, either as the original compound or its metabolites. These residues may result from non-compliance with drug withdrawal periods and incorrect dose protocols [34]. NSAID residues may harm the renal, gastrointestinal, and hematologic systems. To mitigate or eradicate the risk of medication residues in animal-derived foods, adherence to the drug residue withdrawal period is essential [1]. This study did not ascertain the withdrawal time of tolfenamic acid in ducks. It is estimated that drugs are entirely (99.9%) eradicated from the body within 10 times the duration of t_1/2λz_ [35]. Based on this information, following IV and oral administration of 2, 4, and 8 mg/kg doses, the complete elimination of tolfenamic acid from the duck’s body requires 17.20–21.30 h, 29.80–30.62 h, and 41.60–46.20 h, respectively. However, since plasma and tissue concentrations and elimination times of drugs may differ, the withdrawal time must be determined so that tolfenamic acid can be consumed safely in ducks without leaving drug residue.

This study, conducted in ducks, has several limitations that need to be considered. First, it did not look into how tolfenamic acid is broken down in the body or how it is eliminated, which are important for understanding how the drug works and how it might build up. Second, the study did not examine whether factors like the duck’s age or sex affect how the drug behaves in their body. It also did not determine how long the drug stays in tissues or how long farmers need to wait before the meat or eggs are safe to eat. Lastly, the study did not test whether tolfenamic acid actually helps reduce pain or inflammation in ducks. Future research should focus on these areas to better understand the drug’s safety, effectiveness, and proper use in veterinary practice.

## 5. Conclusions

This study is the first to describe the dose-dependent pharmacokinetics of tolfenamic acid in ducks. Increasing doses of tolfenamic acid decreased Cl_T_ and increased t_1/2ʎz_ and AUC with no effect on V_dss_. The drug exhibited high plasma protein binding (99.75%) that is unaffected by concentration. Notably, the commonly used 2 mg/kg dose appears insufficient to achieve therapeutic plasma concentrations, suggesting that dose escalation may be necessary to enhance anti-inflammatory and analgesic efficacy. However, increasing doses could approach saturation of metabolic pathways, risking drug accumulation and potential toxicity if not carefully managed. To translate these findings into clinical practice, it is imperative to develop optimized dosing regimens that balance efficacy and safety. Future research should focus on integrating pharmacokinetic and pharmacodynamic modeling (PK/PD) to identify the minimal effective dose and the maximum tolerated dose across various clinical conditions, such as differing inflammation severity or treatment duration. Additionally, investigations into tissue distribution, safety profiles at higher doses, and in vivo efficacy are essential. By integrating detailed pharmacokinetic information with clinical results, we can develop clear, evidence-based dosing guidelines. This will help veterinarians provide safer and more effective treatments. Using this data-driven approach will make it easier to apply these findings in real-world veterinary practice, leading to better health outcomes for animals.

## Figures and Tables

**Table 1:** Pharmacokinetic parameters of tolfenamic acid following single intravenous and oral administration at doses of 2, 4, and 8 mg/kg in Pekin ducks (*n* = 6).

Parameter		2 mg/kg	4 mg/kg	8 mg/kg
***Intravenous***				
t_1/2ʎz_	h	1.72 (1.62–1.84) ^c^	2.98 (2.78–3.15) ^b^	4.16 (3.97–4.34) ^a^
AUC_0-last_	h *µg/mL	12.90 (10.67–17.02) ^c^	38.23 (30.52–50.17) ^b^	128.44 (100.72–155.83) ^a^
AUC_0-∞_	h *µg/mL	13.03 (10.85–17.19) ^b^	38.45 (30.69–50.51) ^b^	128.76 (100.96–156.29) ^a^
AUC_extrap_	%	1.02 (0.76–1.63)	0.57 (0.48–0.70)	0.25 (0.18–0.32)
MRT_0-∞_	h	1.94 (1.68–2.11) ^c^	2.94 (2.74–3.45) ^b^	3.87 (3.63–4.36) ^a^
Cl_T_	L/h/kg	0.15 (0.12–0.18) ^c^	0.10 (0.80–0.13) ^b^	0.06 (0.05–0.08) ^a^
V_dss_	L/kg	0.30 (0.24–0.39)	0.31 (0.27–0.36)	0.24 (0.21–0.29)
E_body_		0.011 (0.009–0.013) ^c^	0.008 (0.006–0.010) ^b^	0.005 (0.004–0.006) ^a^
***Oral***				
t_1/2ʎz_	h	2.13 (1.90–2.37) ^c#^	3.62 (3.34–3.98) ^b^*	4.62 (4.48–4.81) ^a†^
AUC_0-last_	h *µg/mL	6.18 (5.71–6.53) ^c#^	19.28 (16.24–24.30) ^b^*	56.34 (44.34–62.41) ^a†^
AUC_0-∞_	h *µg/mL	6.32 (5.89–6.70) ^c#^	19.52 (16.46–24.58) ^b^*	56.66 (44.60–62.80) ^a†^
AUC_extrap_	%	2.32 (1.79–2.96)	1.19 (0.92–1.56)	0.56 (0.48–0.62)
MRT_0-∞_	h	3.14 (2.79–3.41) ^c^	4.44 (4.06–5.08) ^b^	5.99 (5.59–6.53) ^a^
MAT	h	1.17 (0.81–1.45)	1.49 (1.19–1.81)	2.11 (1.89–2.39)
T_max_ (median)	h	1.00 (0.75–1.00) ^b^	0.63 (0.25–1.00) ^a^	0.63 (0.25–0.75) ^a^
C_max_	µg/mL	2.25 (2.02–2.48) ^b^	5.94 (5.56–6.20) ^a^	11.40 (10.46–13.60) ^a^
F	%	48.52 (39.00–56.18)	50.77 (48.66–53.93)	44.00 (40.18–51.77)

Note: Data were reported as the geometric mean (min-max). ^a,b,c^: Shows the statistical difference in the same row (*p* < 0.05). ^#,^*^,†^: Shows the difference in these parameters in the same dose group according to IV administration (*p* < 0.05). AUC, area under the concentration-versus-time curve; AUC_extrap_ %, area under the plasma concentration–time curve extrapolated from t_last_ to ∞ in % of the total AUC; Cl_T_, total body clearance; C_max_, peak plasma concentration; E_body_, body extraction ratio; F, bioavailability; MRT_0-∞_, mean residence time; MAT, mean absorption time; t_1/2λz_, terminal elimination half-life; T_max_, time to reach peak plasma concentration; V_dss_, volume of distribution at steady state.

**Table 2:** Plasma protein binding ratio of tolfenamic acid in Pekin ducks.

Tolfenamic Acid Concentration (μg/mL)	Binding Ratio (%)
0.4	99.56 ± 0.08
2	99.83 ± 0.01
10	99.85 ± 0.02
40	99.73 ± 0.35
Mean ± SD	99.74 ± 0.20

Note: No difference between concentration groups (*p* > 0.05).

## Data Availability

The data presented in this study are available upon request from the corresponding author.

## References

[1] Corum O., Uney K., Coskun D., Durna Corum D., Cetin G., Elmas M. (2024). Plasma and milk pharmacokinetics and estimated milk withdrawal time of tolfenamic acid in lactating sheep. Vet. Med. Sci..

[2] CVMP Committee for Veterinary Medicinal Products: Tolfenamic Acid Summary Report; EMEA/MRL/183/97-FINAL. European Medicinces Agency (EMEA). https://www.ema.europa.eu/en/documents/mrl-report/tolfenamic-acid-summary-report-committee-veterinary-medicinal-products_en.pdf.

[3] Ahmed S., Sheraz M.A., Ahmad I. (2018). Tolfenamic acid. Profiles Drug Subst. Excip. Relat. Methodol..

[4] Lees P., McKellar Q.A., Foot R., Gettinby G. (1998). Pharmacodynamics and pharmacokinetics of tolfenamic acid in ruminating calves: Evaluation in models of acute inflammation. Vet. J..

[5] Landoni M.F., Cunningham F.M., Lees P. (1996). Pharmacokinetics and pharmacodynamics of tolfenamic acid in calves. Res. Vet. Sci..

[6] Sidhu P.K., Landoni M.F., Lees P. (2006). Pharmacokinetic and pharmacodynamic interactions of tolfenamic acid and marbofloxacin in goats. Res. Vet. Sci..

[7] Machin K. (2005). Controlling avian pain. Compend. Contin. Educ. Pract. Vet. N. Am. Ed..

[8] Durna Corum D., Corum O., Uney K., Turk E., Sakin F., Giorgi M. (2025). Pharmacokinetics of tolfenamic acid in ducks (*Anas platyrhynchos domestica*) after different administration routes. Br. Poult. Sci..

[9] Hatt J.M., Kreyenbuhl K., Kummrow M. (2023). Methods of analgesia and euthanasia in backyard poultry. Schweiz. Arch. Tierheilkd..

[10] Baert K. (2003). Pharmacokinetics and Pharmacodynamics of Non-Steroidal Anti-Inflammatory Drugs in Birds. Ph.D. Thesis.

[11] Malik A., Valentine A. (2018). Pain in birds: A review for veterinary nurses. Vet. Nurs. J..

[12] Olsen G.H. (2009). Bacterial and parasitic diseases of anseriformes. Vet. Clin. N. Am. Exot. Anim. Pract..

[13] Food and Agriculture Organization (FAO). https://www.fao.org/poultry-production-products/production/poultry-species/en/.

[14] Corum O., Coskun D., Karahan M., Durna Corum D. (2024). Effect of different doses of tolfenamic acid administration to ducks on biochemical parameters. Harran Univ. J. Vet. Med..

[15] Zhou J., Yu J.Z., Zhu M.Y., Yang F.X., Hao J.P., He Y., Zhu X.L., Hou Z.C., Zhu F. (2025). Optimizing breeding strategies for pekin ducks using genomic selection: Genetic parameter evaluation and selection progress analysis in reproductive traits. Appl. Sci..

[16] McKellar Q.A., Lees P., Gettinby G. (1994). Pharmacodynamics of tolfenamic acid in dogs. Evaluation of dose response relationships. Eur. J. Pharmacol..

[17] Corum O., Corum D.D., Er A., Yildiz R., Uney K. (2018). Pharmacokinetics and bioavailability of tolfenamic acid in sheep. J. Vet. Pharmacol. Ther..

[18] Tekeli I.O., Turk E., Durna Corum D., Corum O., Kirgiz F.C., Uney K. (2020). Effect of dose on the intravenous pharmacokinetics of tolfenamic acid in goats. J. Vet. Pharmacol. Ther..

[19] European Medicines Agency (EMA) EMEA/CHMP/EWP/192217/2009. https://www.ema.europa.eu/en/documents/other/overview-comments-received-guideline-validation-bioanalytical-methods-emeachmpewp1922172009_en.pdf.

[20] Cetin G., Corum O., Durna Corum D., Atik O., Altan F., Turk E., Tekeli I.O., Eser Faki H., Uney K. (2022). Pharmacokinetics of intravenous meloxicam, ketoprofen and tolfenamic acid in chukar partridge (*Alectoris chukar*). Br. Poult. Sci..

[21] Corum O., Durna Corum D., Marin P., Acar O.F., Aksoy M., Uney K. (2024). Pharmacokinetics, bioavailability and plasma protein binding of tolfenamic acid in rainbow trout (*Oncorhynchus mykiss*). Vet. Med. Sci..

[22] Turk E., Tekeli I.O., Durna Corum D., Corum O., Sakin F., Uney K. (2021). Pharmacokinetics of tolfenamic acid after different administration routes in geese (*Anser cygnoides*). J. Vet. Pharmacol. Ther..

[23] Dinakaran V., Dumka V.K. (2013). Pharmacokinetics of tolfenamic acid following two oral dose levels in buffalo calves. J. Vet. Pharmacol. Ther..

[24] Lefebvre H.P., Laroute V., Alvinerie M., Schneider M., Vinclair P., Braun J.P., Toutain P.L. (1997). The effect of experimental renal failure on tolfenamic acid disposition in the dog. Biopharm. Drug Dispos..

[25] Laut S., Poapolathep S., Khidkhan K., Klangkaew N., Phaochoosak N., Wongwaipairoj T., Giorgi M., Escudero E., Marin P., Poapolathep A. (2025). Pharmacokinetic characteristics of tolfenamic acid in freshwater crocodiles (*Crocodylus siamensis*). Animals.

[26] Raweewan N., Chomcheun T., Laovechprasit W., Jongkolpath W., Klangkaew N., Phaochoosak N., Poapolathep S. (2020). Pharmacokinetics of tolfenamic acid in green sea turtles (*Chelonia mydas*) after intravenous and intramuscular administration. J. Vet. Pharmacol. Ther..

[27] Raweewan N., Laovechprasit W., Giorgi M., Chomcheun T., Klangkaew N., Imsilp K., Poapolathep S. (2020). Pharmacokinetics of tolfenamic acid in Hawksbill turtles (*Eretmochelys imbricata*) after single intravenous and intramuscular administration. J. Vet. Pharmacol. Ther..

[28] Toutain P.L., Bousquetmélou A. (2004). Plasma clearance. J. Vet. Pharmacol. Ther..

[29] Kuninaka T., Sugai K., Saito T., Mori N., Kimura R., Murata T. (1981). Studies on the metabolism of N-(3-chloro-2-methylphenyl) anthranilic acid (GEA 6414), a new anti-inflammatory agent. I. Urinary metabolites of GEA 6414 in human, dogs, rabbits and rats (author’s transl). Yakugaku Zasshi.

[30] Bennett W.M., Henrich W.L., Stoff J.S. (1996). The renal effects of nonsteroidal anti-inflammatory drugs: Summary and recommendations. Am. J. Kidney Dis..

[31] Dorrestein G.M. (1991). The pharmacokinetics of avian therapeutics. Vet. Clin. N. Am. Small Anim. Pract..

[32] Wasfi I.A., ElGhazali M., Hadi A.A., Zorob O., Boni N.S., Alkatheeri N.A., Barezaiq I.M. (1998). Pharmacokinetics of tolfenamic acid and its detection time in urine after intravenous administration of the drug in camels (*Camelus dromedarius*). Am. J. Vet. Res..

[33] Yáñez J.A., Remsberg C.M., Sayre C.L., Forrest M.L., Davies N.M. (2011). Flip-flop pharmacokinetics--delivering a reversal of disposition: Challenges and opportunities during drug development. Ther. Deliv..

[34] Pratiwi R., Ramadhanti S.P., Amatulloh A., Megantara S., Subra L. (2023). Recent advances in the determination of veterinary drug residues in food. Food.

[35] Brown S.A., Adams H.R. (2001). Pharmacokinetics: Disposition and fate of drugs in the body. Veterinary Pharmacology and Therapeutics.

